# Cellular ROS and Antioxidants: Physiological and Pathological Role

**DOI:** 10.3390/antiox13050602

**Published:** 2024-05-14

**Authors:** Andrey V. Kozlov, Sabzali Javadov, Natascha Sommer

**Affiliations:** 1 Ludwig Boltzmann Institute for Traumatology, The Research Center in Cooperation with AUVA, 1200 Vienna, Austria; 2 Department of Physiology, School of Medicine, University of Puerto Rico, San Juan, PR 00936-5067, USA; 3 Excellence Cluster Cardio-Pulmonary Institute, Universities of Giessen and Marburg Lung Center, Member of the German Center for Lung Research, Justus-Liebig-University, 35392 Giessen, Germany

## 1. Introduction

**Figure 1:**
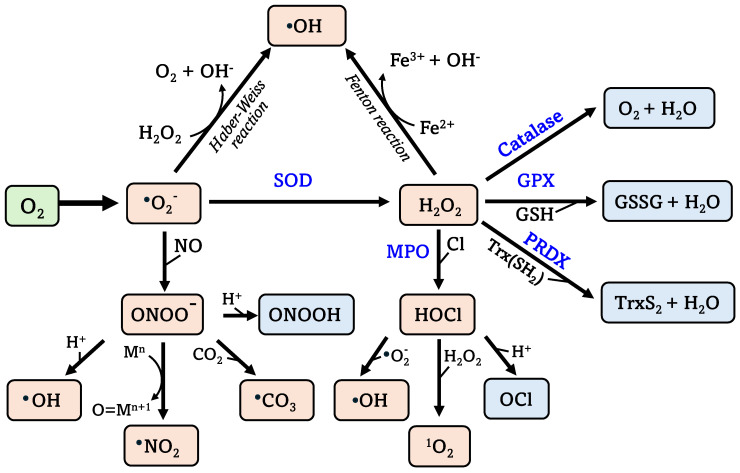
The main ROS generation pathways in cellular environments. *See text for details.* Abbreviations: GPX, glutathione peroxidase; GSH, reduced glutathione; GSSG, glutathione disulfide; M(n) and O = M(n + 1), reduced and oxidized metalloprotein; MPO, myeloperoxidase; PRDX, peroxiredoxin; SOD, superoxide dismutase; Trx(SH2) and TrxS2, reduced and oxidized thioredoxin.

Reactive oxygen species (ROS) are highly reactive oxygen derivatives that include free radicals such as superoxide anion radical (O_2_^•−^) and hydroxyl radical (HO^•^), as well as non-radical molecules hydrogen peroxide (H_2_O_2_), peroxynitrite (ONOO^−^), and hypochlorous acid (HOCl). ROS interact with each other to produce new radicals exhibiting varying reactivity and properties within living systems. Among ROS, O_2_^•−^ is the primary oxygen radical produced through the one-electron oxygen reduction. Superoxide dismutase (SOD) converts O_2_^•−^ into hydrogen peroxide (H_2_O_2_), serving as a precursor for other ROS. Through the Haber-Weiss reaction, O_2_^•−^ generates HO^•^, a highly reactive ROS within the cell. Similarly, H_2_O_2_ produces HO^•^ through Fenton reactions in the presence of iron. Interaction between O_2_^•−^ and nitric oxide (NO) leads to ONOO-formation, elevating reactive nitrogen species (RNS) levels. RNS, derived from nitric oxide, regulate metabolic and functional pathways in various cells, including smooth muscle cells, cardiomyocytes, platelets, and nervous and juxtaglomerular cells [1]. However, under pathological conditions, elevated RNS levels induce nitrosative stress, leading to cell injury and death. In addition, H_2_O_2_ generates the strong oxidant HOCl through myeloperoxidase (MPO)-mediated chlorine (Cl) peroxidation. Under physiological conditions, a robust antioxidant system comprising enzymatic and non-enzymatic pathways maintains a tightly regulated balance between ROS generation and elimination (Figure 1).

**Figure 2:**
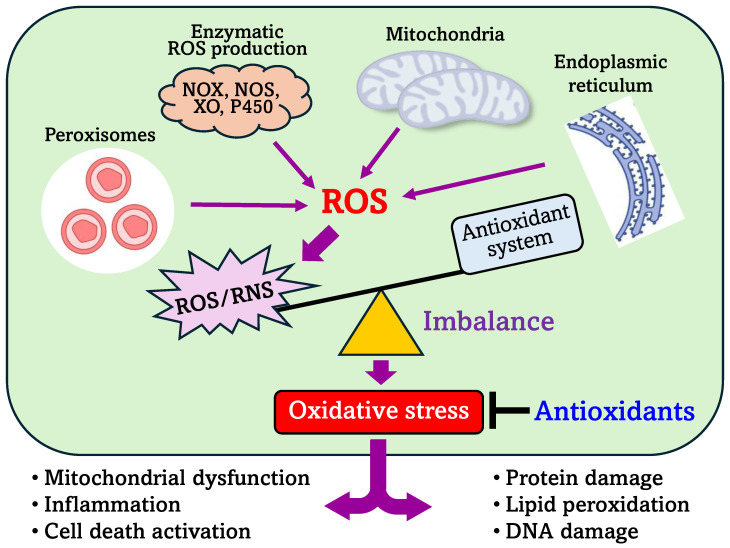
Intracellular sources of ROS and consequences of oxidative stress. *See text for details.* Abbreviations: NOS, nitric oxide synthase; NOX, NADPH oxidases; XO, xanthine oxidase.

ROS are generated within cells in various compartments, including the cytoplasm and subcellular organelles such as mitochondria, peroxisomes, and the endo(sarco)plasmic reticulum. Mitochondria play a crucial role in cell life, influencing the development of nearly all human diseases and contributing to the aging process [2]. Mitochondria are a major source of ROS in mammalian cells, with ROS production occurring at more than ten different sites within these organelles. These sites include complexes within the electron transport chain, iron-sulfur clusters, the α-ketoglutarate dehydrogenase complex (α-KGDH), mitochondrial P450 systems, and monoamine oxidase (MAO), among others. In the cytoplasm, peroxisomes, and the endo(sarco)plasmic reticulum, ROS generation is facilitated by various enzymes and metabolic pathways. This includes reactions catalyzed by nitric oxide synthases (NOS), NADPH oxidases (NOX), xanthine oxidase (XO), D-amino acid oxidase (DAO), enzymes involved in fatty acid β-oxidation, flavoenzymes, cytochrome P450 enzymes, and other metabolic pathways. These diverse sources collectively contribute to the cellular pool of ROS (Figure 2).

Maintaining a balance between ROS production and antioxidant defense mechanisms is crucial for cellular health. Cells possess a robust antioxidant defense system in the cytoplasm and cellular organelles, comprising enzymatic and non-enzymatic antioxidants that synergize ROS and uphold redox balance. Essential enzymatic antioxidants include SOD, catalase, glutathione peroxidase, and glutathione reductase. Non-enzymatic antioxidants include molecules like glutathione (GSH), ubiquinone (coenzyme Q_10_), vitamins C and E, alpha-lipoic acid, and thioredoxins. These antioxidants counteract excess ROS, mitigating oxidative stress and preserving cellular redox homeostasis. Imbalances that favor ROS over antioxidant capacity can trigger oxidative damage, initiating pathological pathways associated with various diseases (Figure 2).

This Special Issue of *Antioxidants*, titled “Cellular ROS and Antioxidants: Physiological and Pathological Roles,” for which we served as Guest Editors, has published 17 articles, consisting of 13 original studies and four review articles. The issue explores the dual nature of ROS, examining both their beneficial and harmful impacts and analyzing the transition between these effects. Moreover, it assesses the advantages and limitations of antioxidant therapy across different scenarios. This Editorial aims not to explore the literature on this topic excessively but to succinctly highlight various aspects of ROS contributions to cell health and damage and the effects of antioxidants. We will primarily refer to relevant reviews to provide a comprehensive overview.

Additionally, we aim to fill any remaining gaps in the field by incorporating contributions from this Special Issue. ROS were considered the side products of cellular metabolism that could induce oxidative damage to biomolecules, leading to cellular dysfunction and death. However, studies over the last 30 years have provided strong evidence that ROS play an essential role in intracellular signaling and regulate many critical cellular functions, such as bactericidal activity, metabolic reaction, and gene expression. It is commonly accepted that mitochondrial ROS play a predominant role in orchestrating ROS generated from other sources and regulating ROS-dependent intracellular metabolism. A combination of extramitochondrial and intramitochondrial factors controls ROS generation within mitochondria. Among these, alterations in matrix volume and mitochondrial swelling mechanisms are primary factors that contribute significantly to the regulation of ROS production. In this Special Issue, a review study by Chapa-Dubocq et al. [3] discusses the mechanisms responsible for mitochondrial volume regulation in cardiomyocytes.

Over the last decade, a growing body of literature indicates that ROS play a role in the body’s physiological and adaptive/defense responses. This suggests that antioxidants might have beneficial and potentially harmful effects if they remove ROS from essential physiological and adaptive pathways. However, compared to ROS, which was exhaustively addressed in the literature, the adverse impact of antioxidants was much less addressed. In this Special Issue, most studies also confirm the beneficial effects of antioxidants, and only a few communications indicate that antioxidants may have adverse effects. Intriguingly, the conclusion about the beneficial effects of antioxidants is generally made in preclinical models, whereas clinical trials show diverse results. The divergent effects are often attributed to the biological heterogeneity among patients and/or variations in study designs between preclinical studies and clinical trials. However, mechanistic reasons for this diversity are seldom provided. In this editorial, we tried to classify the beneficial and adverse effects of antioxidants and the relation of the contributions in this Special Issue to these diverse actions of antioxidants. Antioxidant activity definitions range from direct scavenging ROS to decreased ROS/oxidation product levels in biological systems. The latter includes ROS scavengers and substances that inhibit ROS reactions, such as iron chelators or inhibitors of specific enzymes producing ROS, such as NOX inhibitors. Here, we classify any action that reduces ROS levels as an antioxidant action.

## 2. Pleiotropic Actions of ROS and Antioxidants

The primary mechanism underlying the beneficial effects of antioxidants is a decrease in ROS levels or oxidation products, which prevents oxidative damage to cells and organs. A central counteracting mechanism is the scavenging of ROS released by immune cells aiming to kill pathogens and antioxidants logically, which can hinder this ability of immune cells, resulting in an accumulation of live bacteria in phagocytic cells. The inability to kill bacteria due to low ROS levels also occurs due to a genetic defect of NOS2, an enzyme generating ROS in phagosomes responsible for developing granulomatous disease. Logically, the inhibition of this enzyme by antioxidants may have a similar deleterious effect. It is, however, questionable if genetic manipulation can be attributed to antioxidant action. Concerning ROS-mediated damage to host cells, those ROS should be generated by the immune cells in the extracellular space; otherwise, they do not come into contact with the host cells. Consequently, upon inflammatory response, it is essential to consider which compartment the antioxidants exert their activity: the extracellular compartment preventing organ damage or the intracellular compartment (phagosome) inhibiting phagocytosis.

An illustrative example is the application of mitochondria-targeted antioxidants in aseptic and septic inflammation. It is known that mitochondrial ROS regulate the activity of NOS [4,5] and consequently attenuate both bactericidal activity and damage to host cells. It has been shown that mitochondria-targeted antioxidants exert opposite effects in two animal models of systemic inflammatory response syndrome (SIRS). They reduced organ damage in the lipopolysaccharide (LPS)-induced SIRS model [6], while in an acute polymicrobial sepsis model, they increased the mortality rate [7]. A significant difference between the two models is that the first one is accompanied by a sterile inflammatory response induced by LPS, and in the second one, the body should combat live pathogens. Mitochondrial ROS and mitochondria-targeted antioxidants modulate the immune system’s efficiency and host cell damage [8], inhibiting ROS generation in intracellular and extracellular compartments. The action of antioxidants can also be organ-specific. For instance, it has been shown that mitoTEMPO has a beneficial effect on the majority of organs due to its antioxidant part (TEMPO). Still, the beneficial impact on the lung is due to phosphonium, the positively charged transport part of mitoTEMPO [9]. This controversy in the action of antioxidants, which depends on different pathological settings, affected signaling pathways, and different body/cell compartments, is often called the pleiotropic effects of antioxidants. A review article by Murai and Matsuda [10] discusses the pleiotropic signaling pathways mediated by ROS, which can simultaneously affect different and even opposite functions. They can regulate cellular redox status regulation and act as second messengers. Conversely, ROS can induce oxidative stress at high concentrations, leading to cell damage, cell death, and disease development. The study mainly focuses on the interaction between ROS, phytochemicals, and gut microbiota in the environment characteristic of cancer [10].

The balance between the beneficial and harmful effects of ROS and RNS depends on the quantity and quality of reactive species. Such superoxide radicals or hydrogen peroxide species are not very aggressive. They are more suitable for signaling reactions based predominantly on the oxidation of SH groups, changing the conformation of specific enzymes and activating them [8,11]. In contrast, peroxynitrite, hydroxyl radical, and hypochlorous acid predominantly damage biological structures [11]. ROS are very short-living molecules, and detecting ROS in living systems is complex and requires new developments. In the present Special Issue, Cubas et al. [12] developed a susceptible H_2_O_2_ sensor capable of quantitatively analyzing the ratio of extracellular peroxides to intracellular peroxides across the plasma membrane. They found that this ratio is approximately 300:1 and remains similar in different species.

To fully consider the interaction between ROS and antioxidants, we attempted to classify the significant beneficial and harmful effects of ROS and antioxidants based on the existing literature and contributions to our Special Issue. We consider that ROS and antioxidants are almost counteracted; if ROS exert a harmful effect, then antioxidant treatment should be beneficial and vice versa, except for antioxidants, which exert a pro-oxidative impact as described below. The primary beneficial effects of antioxidants addressed in the literature include the following actions.

## 3. Deleterious Effects of ROS Mitigated by Antioxidants

### 3.1. Antioxidants Reduce the Risk of Chronic Diseases

A growing body of studies suggests that by neutralizing harmful free radicals, antioxidants prevent oxidative damage to cells and tissues, which are implicated in developing chronic diseases. Antioxidants play a crucial role in reducing the risk of chronic liver diseases [13], chronic heart failure [14], neurodegenerative diseases [15,16], and chronic kidney disease [17]. It has also been shown that antioxidants can reduce cardiotoxicity induced by anticancer therapy [18]. The intracellular ROS pool is not homogeneous; ROS released from specific sources or compartments can be responsible for specific harmful effects. Conversely, inhibition of these particular sources (processes) of ROS generation can exert beneficial effects against cell/tissue damage. In this Special Issue, Casado-Barragán et al. [19] showed that NOX-4 is a primary source of ROS responsible for the induction of profibrotic factors in the renal medulla. Consequently, these factors cause chronic kidney disease differently regulated in male and female mice and can be alleviated by specific NOX4 inhibitors.

### 3.2. Antioxidants Protect against Aging

Oxidative stress is commonly recognized as one of the main factors contributing to aging [20]. Antioxidants have been shown to prevent oxidative damage in cell aging [21]. Antioxidant treatment has demonstrated the ability to preserve skin health during aging [22]. In this Special Issue, Hui et al. [23] showed that the heat shock 70-kDa protein 8 (HSPA8) could regulate senescence in fibroblasts exposed to oxygen-glucose deprivation through interaction with ALDH2, the activity of which is associated with ROS levels in the cells. This study is vital for developing new directions for effective treatment of fibroblast senescence after myocardial infarction. In contrast to aging, antioxidant supplementation therapies for aging-associated diseases reported controversial outcomes [24]. The latter was associated with the opposite action of antioxidants due to patient-specific metabolic demand and genetic background.

### 3.3. Antioxidants Improve Immune Therapy

Antioxidants support a healthy immune system by protecting host cells, especially immune cells, from oxidative damage, thereby reducing organ failure and other adverse health effects [25]. These effects indicate that antioxidants support optimal immune function, enhancing the body’s ability to prevent cell damage from immune responses. It must also engage in the necessary fight against pathogens that trigger ROS production. Thus, there could be a conflict that results in the application of antioxidants upon infection and immune therapy.

### 3.4. Antioxidants Protect against Cardiovascular and Respiratory Disorders

Antioxidants such as flavonoids and polyphenols found in fruits, vegetables, and other plant-based foods are associated with a reduced risk of cardiovascular diseases, such as ischemic heart disease [26]. They help to improve cardiac function by protecting against oxidative damage to blood vessels [27], lowering vessel wall inflammation, and improving cholesterol levels [28]. Furthermore, recently developed mitochondria-targeted antioxidants with ROS and electron scavenging capacity, such as XJB-5-131, have been shown to protect against cardiac ischemia-reperfusion injury in adult [29] and aged [30] rats, improve aging-associated skeletal muscle contractility [31], and exhibit anti-ferroptotic effects in cardiomyocytes [32]. In this Special Issue, a comprehensive review article by Lim et al. [33] discusses the beneficial effects of a large spectrum of antioxidants in acute respiratory distress syndrome (ARDS). The study emphasizes the potential benefits of using antioxidants to inhibit ROS accumulation as a promising therapeutic strategy for patients with ARDS. It also underscores the critical need for additional clinical trials to evaluate the therapeutic effectiveness of antioxidants in this context.

### 3.5. Antioxidants Prevent Oxidative Stress in Kidney Transplantation and Intestinal Angiogenesis

Oxidative stress plays a crucial role in organ transplantation and intestinal angiogenesis, although the mechanisms underlying these conditions and the effects of antioxidants remain unknown. In this Special Issue, a review article by Granata et al. [34] summarizes all relevant knowledge on the role of oxidative stress and ischemia-reperfusion injury in kidney transplantation, mainly focusing on ferroptosis and mitophagy and describing the potential beneficial effects of new antioxidants. Wang et al. [35] reported the nephroprotective effect of dioscin, an antioxidant and anti-inflammatory drug, during kidney transplantation. The beneficial effects of dioscin against cisplatin-induced acute kidney injury were mediated by reducing ferroptosis and apoptosis through activating the Nrf2/HO-1 signaling pathway. In another contribution by Zou et al. [36], oxidative stress induced the upregulation of dual oxidase 2 (DUOX2) in the intestine of low-birth-weight piglets, whereas DUOX2 knockdown decreased ROS levels and increased the angiogenesis in a matrix metalloproteinase 3 (MMP3)-dependent manner. These findings suggest that DUOX2-induced oxidative stress inhibited intestinal angiogenesis through MMP3 in piglets.

### 3.6. Antioxidants and Eye Health

Antioxidants such as lutein, zeaxanthin [37], and vitamin C [38] have been shown to confer significant benefits to ocular health, contributing to maintaining healthy eyes. These antioxidants play crucial roles in preventing oxidative stress within ocular tissues, thereby helping to preserve visual function and protect against age-related degenerative changes and cataracts [39]. Incorporating these antioxidants into therapeutic regimens supports ocular health and underscores their potential as preventive measures against eye-related conditions.

### 3.7. Antioxidants and Brain Health

Antioxidants have neuroprotective properties and may help to prevent or delay the onset of neurodegenerative diseases by reducing oxidative stress in the brain [40]. In this Special Issue, Jovanovic et al. [41] reported a complex interaction between ROS and antioxidants in a model of Parkinson’s disease (PD). This study demonstrated that intermittent theta burst stimulation (iTBS) in a rat model of PD significantly bolsters antioxidative capacity and acts as a neuroprotective mechanism in specific brain regions associated with PD. Some antioxidants, such as flavonoids and omega-3 fatty acids, have been linked to improved cognitive function and a reduced risk of conditions like dementia and Alzheimer’s disease [42]. In this Special Issue, U-Pathi et al. [43] showed that aspartame-induced neurotoxicity in the cerebral cortex of rats is driven by ROS resulting from the interplay between inflammation, enhanced oxidant stress, decreased mitochondrial biogenesis, and apoptosis. This ROS-mediated mechanism suggests the beneficial effects of antioxidants against aspartame-induced neurotoxicity. In addition, Kim et al. [44] showed that epigallocatechin-3-gallate (EGCG), a clinically relevant antioxidant, attenuates neuronal death, accompanied by GPX1 induction and preserved mitochondria in neurons by activating the ERK1/2. MAPK.

### 3.8. Antioxidants Promote Skin Health

Antioxidants have been shown to protect the skin against damage caused by UV radiation, environmental pollutants, and other stressors [45]. They can decrease inflammation, delay premature aging (such as wrinkles and fine lines), and enhance skin texture and appearance. Common antioxidants in skincare formulations include vitamins C and E and coenzyme Q_10_ [46].

### 3.9. Anti-Inflammatory Effects of Antioxidants

Many antioxidants also have anti-inflammatory properties, which can help reduce inflammation in the body. They are associated with various health problems, including cardiac diseases, brain injury, diabetes, and autoimmune disorders, so reducing inflammation can have significant health benefits. In this Special Issue, Aguida et al. [47] reported how near-infrared light influences the balance between pro- and anti-inflammatory cytokines by regulating mitochondrial ROS production. In addition, Song et al. [48] demonstrated the antioxidant capacity of *Artemisia gmelinii* extract (AGE). This natural product suppresses neutrophil infiltration and lung damage by inhibiting the NF-κB/MAPK and enhancing the NRF2/HO-1 signaling pathways.

### 3.10. Antioxidants and Cancer Prevention

Although further research is necessary, some studies propose that antioxidants may lower the risk of specific types of cancer by protecting cells from DNA damage and inhibiting the growth of cancerous cells. However, numerous clinical trials, including β-carotene, vitamin E, vitamin C, selenium, retinol, zinc, riboflavin, and molybdenum, did not produce convincing evidence to justify the use of antioxidants for cancer prevention [49].

### 3.11. Antioxidants Improve Exercise Performance

Certain antioxidants, like vitamins C and E, can enhance exercise performance and help in recovery by diminishing oxidative stress and mitigating muscle damage caused by intense physical activity. Athletes and active individuals may benefit from antioxidant supplementation to support their training regimen. Recent studies have indicated that the beneficial effects of antioxidants primarily stem from natural sources in food rather than from antioxidant supplements [50]. This raises questions about the combined effects of antioxidants with other nutrients present in the food, which may facilitate the beneficial effects of natural antioxidants. However, the literature has not thoroughly explored the interaction between antioxidants and other nutrients.

## 4. Navigating the Paradox of Antioxidants: Unveiling Deleterious Effects

Most of the literature on the biological action of antioxidants highlights their beneficial effects or reports the absence of adverse effects. However, a smaller portion of the literature also documents deleterious effects. Here, we will describe the studies in our Special Issue that discuss the harmful effects of antioxidants.

### 4.1. Antioxidants Exert Pro-Oxidant Activity

Antioxidants can act as pro-oxidants, which may generate free radicals, potentially exacerbate oxidative stress, and damage cellular components under certain conditions. This paradoxical behavior has been observed in studies examining high doses of certain antioxidants, such as vitamins C and E. Surprisingly, some popular antioxidants have been reported to have pro-oxidant behavior. At least three factors can influence the function of an antioxidant, transforming it into a pro-oxidant; these factors include the presence of metal ions, the concentration of the antioxidant in matrix environments, and its redox potential [51]. The most illustrative example is ascorbic acid, which, on the one hand, can recycle antioxidants to their active form but, on the other hand, exhibits predominantly pro-oxidant activity in the presence of iron ions, reducing Fe^3+^ (ferric ion) to Fe^2+^ (ferrous ion) [52]. It has been reported that the consumption of antioxidant supplements produces dose-related effects, with beneficial effects occurring at low doses and adverse effects occurring when high doses of antioxidants are consumed [53]. The latter aligns with the conditions required for the pro-oxidant activity of antioxidants.

### 4.2. Antioxidants Increase the Risk of Cancer and Weaken Anticancer Therapy

Contrary to popular belief, some studies have suggested that excessive antioxidant supplementation may increase the risk of cancer. For example, high doses of beta-carotene [54] and vitamin E [55] supplements have been associated with an elevated risk of lung cancer and prostate cancer, respectively. Antioxidant supplementation also alters the efficacy of anticancer therapy, which is often based on oxidative damage of the cancer cells [56]. In combination with anticancer therapy, antioxidants may protect cancer cells from oxidative stress-induced cell death, allowing them to proliferate and metastasize more effectively.

### 4.3. Antioxidants Interfere with Cell Signaling

The body maintains a delicate balance between ROS and antioxidants, known as redox balance. Excessive intake of antioxidants can disrupt this balance, leading to a condition known as reductive stress. The latter impairs cellular signaling pathways, interfering with normal physiological processes. ROS serve essential roles in cell signaling processes; consequently, excessive neutralization of ROS by antioxidants can disrupt these signaling processes, affecting pathways mediated by intracellular messengers, such as NO, CO, H_2_S, and the recently described CH4. In this Special Issue, Keppler et al. [57] showed the capability of eukaryotic cells to synthesize CH4 via a radical-driven process. In addition, Horváth et al. [58] demonstrated that although CH4 is synthesized in a radical-dependent way, it protects mitochondrial function and maintains Ca^2+^ homeostasis. These data suggest that antioxidants can affect physiological CH4-mediated signaling.

### 4.4. Antioxidants Compromise Immune Function

ROS are crucial in the immune system’s response to infections and pathogens. By neutralizing ROS, antioxidants may impair the immune system’s ability to combat microbial pathogens effectively. This can potentially compromise the body’s defense mechanisms against infections. A review study by Zhang et al. [59] in this Special Issue summarizes the current understanding. It discusses the role of the oxidative damage repair pathway mediated by 8-oxoG DNA glycosylase 1 (OGG1) in activating and maintaining immune cell function. It has been shown that bilirubin impairs the bactericidal activity of neutrophils through an antioxidant mechanism [60]. In this Special Issue, Tsai et al. [61] showed that the mitochondria-targeted antioxidants Mito-TEMPO and MCC950 increased caspase-1p20 and IL-1β expression in THP-1 macrophages. These findings indicate that mitochondrial ROS and autophagy could interrupt THP-1-M1 macrophage polarization, compromising the function of the immune system. The latter can impair the function of the immune system. The application of mitoTEMPO reduces the survival rate in rats subjected to the CLP model of sepsis [7]. Thus, antioxidants can protect tissues against an excessive inflammatory response but simultaneously can weaken the immune system [8].

## 5. Conclusions

ROS and antioxidants are involved in pathological, physiological, and related defense system processes. This shows that the redox balance regulated by ROS and antioxidants defines whether antioxidants are beneficial, indifferent, or harmful. This balance is not static and depends on the pathophysiological status of the body, the compartment where ROS are produced, and the type of ROS. The redox status benefits a healthy body, but it may be harmful under specific pathological circumstances and vice versa. An important issue is the compartmentalization of the action of ROS and antioxidants. Elevation or inhibition of ROS in different compartments can have the opposite biological effects. Compartmentalization of ROS generation, allocated to specific compartments enriched with a corresponding ROS generating system (e.g., NOX or mitochondria), is well known. In contrast, we still do not know much about the compartment-specific action of most natural antioxidants. However, more is known about synthetic mitochondria-targeted antioxidants and specific inhibitors of ROS-generating enzymes. The compartment-specific action of antioxidants seems to be the limiting factor for understanding their biological effects on the body. Further research is required to address this question, particularly under pathological circumstances.

## References

[1] Martinez M.C., Andriantsitohaina R. (2009). Reactive nitrogen species: Molecular mechanisms and potential significance in health and disease. Antioxid. Redox Signal..

[2] Javadov S., Kozlov A.V., Camara A.K.S. (2020). Mitochondria in Health and Diseases. Cells.

[3] Chapa-Dubocq X.R., Rodriguez-Graciani K.M., Escobales N., Javadov S. (2023). Mitochondrial Volume Regulation and Swelling Mechanisms in Cardiomyocytes. Antioxidants.

[4] Daiber A., Di Lisa F., Oelze M., Kroller-Schon S., Steven S., Schulz E., Munzel T. (2017). Crosstalk of mitochondria with NADPH oxidase via reactive oxygen and nitrogen species signalling and its role for vascular function. Br. J. Pharmacol..

[5] Dikalova A., Clempus R., Lassegue B., Cheng G., McCoy J., Dikalov S., San Martin A., Lyle A., Weber D.S., Weiss D. (2005). Nox1 overexpression potentiates angiotensin II-induced hypertension and vascular smooth muscle hypertrophy in transgenic mice. Circulation.

[6] Weidinger A., Mullebner A., Paier-Pourani J., Banerjee A., Miller I., Lauterbock L., Duvigneau J.C., Skulachev V.P., Redl H., Kozlov A.V. (2015). Vicious inducible nitric oxide synthase-mitochondrial reactive oxygen species cycle accelerates inflammatory response and causes liver injury in rats. Antioxid. Redox Signal..

[7] Rademann P., Weidinger A., Drechsler S., Meszaros A., Zipperle J., Jafarmadar M., Dumitrescu S., Hacobian A., Ungelenk L., Rostel F. (2017). Mitochondria-Targeted Antioxidants SkQ1 and MitoTEMPO Failed to Exert a Long-Term Beneficial Effect in Murine Polymicrobial Sepsis. Oxidative Med. Cell. Longev..

[8] Kozlov A.V., Lancaster J.R., Meszaros A.T., Weidinger A. (2017). Mitochondria-meditated pathways of organ failure upon inflammation. Redox Biol..

[9] Weidinger A., Birgisdottir L., Schaffer J., Meszaros A.T., Zavadskis S., Mullebner A., Hecker M., Duvigneau J.C., Sommer N., Kozlov A.V. (2022). Systemic Effects of mitoTEMPO upon Lipopolysaccharide Challenge Are Due to Its Antioxidant Part, While Local Effects in the Lung Are Due to Triphenylphosphonium. Antioxidants.

[10] Murai T., Matsuda S. (2023). Pleiotropic Signaling by Reactive Oxygen Species Concerted with Dietary Phytochemicals and Microbial-Derived Metabolites as Potent Therapeutic Regulators of the Tumor Microenvironment. Antioxidants.

[11] Weidinger A., Kozlov A.V. (2015). Biological Activities of Reactive Oxygen and Nitrogen Species: Oxidative Stress versus Signal Transduction. Biomolecules.

[12] de Cubas L., Mallor J., Herrera-Fernandez V., Ayte J., Vicente R., Hidalgo E. (2023). Expression of the H_2_O_2_ Biosensor roGFP-Tpx1.C160S in Fission and Budding Yeasts and Jurkat Cells to Compare Intracellular H_2_O_2_ Levels, Transmembrane Gradients, and Response to Metals. Antioxidants.

[13] Zhang C.Y., Liu S., Yang M. (2023). Antioxidant and anti-inflammatory agents in chronic liver diseases: Molecular mechanisms and therapy. World J. Hepatol..

[14] Gao L., Wang W., Liu D., Zucker I.H. (2007). Exercise training normalizes sympathetic outflow by central antioxidant mechanisms in rabbits with pacing-induced chronic heart failure. Circulation.

[15] Fields M., Marcuzzi A., Gonelli A., Celeghini C., Maximova N., Rimondi E. (2023). Mitochondria-Targeted Antioxidants, an Innovative Class of Antioxidant Compounds for Neurodegenerative Diseases: Perspectives and Limitations. Int. J. Mol. Sci..

[16] Moren C., deSouza R.M., Giraldo D.M., Uff C. (2022). Antioxidant Therapeutic Strategies in Neurodegenerative Diseases. Int. J. Mol. Sci..

[17] Small D.M., Coombes J.S., Bennett N., Johnson D.W., Gobe G.C. (2012). Oxidative stress, anti-oxidant therapies and chronic kidney disease. Nephrology.

[18] Mendez-Valdes G., Gomez-Hevia F., Bragato M.C., Lillo-Moya J., Rojas-Sole C., Saso L., Rodrigo R. (2023). Antioxidant Protection against Trastuzumab Cardiotoxicity in Breast Cancer Therapy. Antioxidants.

[19] Casado-Barragan F., Lazcano-Paez G., Larenas P.E., Aguirre-Delgadillo M., Olivares-Aravena F., Witto-Oyarce D., Nunez-Allimant C., Silva K., Nguyen Q.M., Cardenas P. (2023). Increased Renal Medullary NOX-4 in Female but Not Male Mice during the Early Phase of Type 1 Diabetes: Potential Role of ROS in Upregulation of TGF-beta1 and Fibronectin in Collecting Duct Cells. Antioxidants.

[20] Iakovou E., Kourti M. (2022). A Comprehensive Overview of the Complex Role of Oxidative Stress in Aging, the Contributing Environmental Stressors and Emerging Antioxidant Therapeutic Interventions. Front. Aging Neurosci..

[21] Terracina S., Petrella C., Francati S., Lucarelli M., Barbato C., Minni A., Ralli M., Greco A., Tarani L., Fiore M. (2022). Antioxidant Intervention to Improve Cognition in the Aging Brain: The Example of Hydroxytyrosol and Resveratrol. Int. J. Mol. Sci..

[22] Tsai T.Y., Lin R.J., Liu C., Tseng Y.P., Chan L.P., Liang C.H. (2022). Djulis supplementation against oxidative stress and ultraviolet radiation-induced cell damage: The influence of antioxidant status and aging of skin in healthy subjects. J. Cosmet. Dermatol..

[23] Hui W., Song T., Yu L., Chen X. (2023). The Binding of HSPA8 and Mitochondrial ALDH2 Mediates Oxygen-Glucose Deprivation-Induced Fibroblast Senescence. Antioxidants.

[24] Conti V., Izzo V., Corbi G., Russomanno G., Manzo V., De Lise F., Di Donato A., Filippelli A. (2016). Antioxidant Supplementation in the Treatment of Aging-Associated Diseases. Front. Pharmacol..

[25] Ajith Y., Dimri U., Dixit S.K., Singh S.K., Gopalakrishnan A., Madhesh E., Rajesh J.B., Sangeetha S.G. (2017). Immunomodulatory basis of antioxidant therapy and its future prospects: An appraisal. Inflammopharmacology.

[26] Bandyopadhyay D., Chattopadhyay A., Ghosh G., Datta A.G. (2004). Oxidative stress-induced ischemic heart disease: Protection by antioxidants. Curr. Med. Chem..

[27] Diaz M.N., Frei B., Vita J.A., Keaney J.F. (1997). Antioxidants and atherosclerotic heart disease. N. Engl. J. Med..

[28] Malekmohammad K., Sewell R.D.E., Rafieian-Kopaei M. (2019). Antioxidants and Atherosclerosis: Mechanistic Aspects. Biomolecules.

[29] Jang S., Lewis T.S., Powers C., Khuchua Z., Baines C.P., Wipf P., Javadov S. (2017). Elucidating Mitochondrial Electron Transport Chain Supercomplexes in the Heart during Ischemia-Reperfusion. Antioxid. Redox Signal..

[30] Escobales N., Nunez R.E., Jang S., Parodi-Rullan R., Ayala-Pena S., Sacher J.R., Skoda E.M., Wipf P., Frontera W., Javadov S. (2014). Mitochondria-targeted ROS scavenger improves post-ischemic recovery of cardiac function and attenuates mitochondrial abnormalities in aged rats. J. Mol. Cell. Cardiol..

[31] Javadov S., Jang S., Rodriguez-Reyes N., Rodriguez-Zayas A.E., Soto Hernandez J., Krainz T., Wipf P., Frontera W. (2015). Mitochondria-targeted antioxidant preserves contractile properties and mitochondrial function of skeletal muscle in aged rats. Oncotarget.

[32] Rodriguez-Graciani K.M., Chapa-Dubocq X.R., Ayala-Arroyo E.J., Chaves-Negron I., Jang S., Chorna N., Maskrey T.S., Wipf P., Javadov S. (2022). Effects of Ferroptosis on the Metabolome in Cardiac Cells: The Role of Glutaminolysis. Antioxidants.

[33] Lim E.Y., Lee S.Y., Shin H.S., Kim G.D. (2023). Reactive Oxygen Species and Strategies for Antioxidant Intervention in Acute Respiratory Distress Syndrome. Antioxidants.

[34] Granata S., Votrico V., Spadaccino F., Catalano V., Netti G.S., Ranieri E., Stallone G., Zaza G. (2022). Oxidative Stress and Ischemia/Reperfusion Injury in Kidney Transplantation: Focus on Ferroptosis, Mitophagy and New Antioxidants. Antioxidants.

[35] Wang S., Zheng Y., Jin S., Fu Y., Liu Y. (2022). Dioscin Protects against Cisplatin-Induced Acute Kidney Injury by Reducing Ferroptosis and Apoptosis through Activating Nrf2/HO-1 Signaling. Antioxidants.

[36] Zou D., Yang Y., Ji F., Lv R., Xu T., Hu C. (2023). DUOX2-Induced Oxidative Stress Inhibits Intestinal Angiogenesis through MMP3 in a Low-Birth-Weight Piglet Model. Antioxidants.

[37] Evans J.R., Lawrenson J.G. (2023). Antioxidant vitamin and mineral supplements for slowing the progression of age-related macular degeneration. Cochrane Database Syst. Rev..

[38] Fleckenstein M., Schmitz-Valckenberg S., Chakravarthy U. (2024). Age-Related Macular Degeneration: A Review. JAMA.

[39] Kushwah N., Bora K., Maurya M., Pavlovich M.C., Chen J. (2023). Oxidative Stress and Antioxidants in Age-Related Macular Degeneration. Antioxidants.

[40] Guerra-Araiza C., Alvarez-Mejia A.L., Sanchez-Torres S., Farfan-Garcia E., Mondragon-Lozano R., Pinto-Almazan R., Salgado-Ceballos H. (2013). Effect of natural exogenous antioxidants on aging and on neurodegenerative diseases. Free Radic. Res..

[41] Zeljkovic Jovanovic M., Stanojevic J., Stevanovic I., Ninkovic M., Nedeljkovic N., Dragic M. (2024). Sustained Systemic Antioxidative Effects of Intermittent Theta Burst Stimulation beyond Neurodegeneration: Implications in Therapy in 6-Hydroxydopamine Model of Parkinson’s Disease. Antioxidants.

[42] Cole G.M., Lim G.P., Yang F., Teter B., Begum A., Ma Q., Harris-White M.E., Frautschy S.A. (2005). Prevention of Alzheimer’s disease: Omega-3 fatty acid and phenolic anti-oxidant interventions. Neurobiol. Aging.

[43] U-pathi J., Yeh Y.C., Chen C.W., Owaga E.E., Hsieh R.H. (2023). Relationship between Aspartame-Induced Cerebral Cortex Injury and Oxidative Stress, Inflammation, Mitochondrial Dysfunction, and Apoptosis in Sprague Dawley Rats. Antioxidants.

[44] Kim J.E., Kim T.H., Kang T.C. (2023). EGCG Attenuates CA1 Neuronal Death by Regulating GPx1, NF-kappaB S536 Phosphorylation and Mitochondrial Dynamics in the Rat Hippocampus following Status Epilepticus. Antioxidants.

[45] Masaki H. (2010). Role of antioxidants in the skin: Anti-aging effects. J. Dermatol. Sci..

[46] Pegoraro N.S., Barbieri A.V., Camponogara C., Mattiazzi J., Brum E.S., Marchiori M.C.L., Oliveira S.M., Cruz L. (2017). Nanoencapsulation of coenzyme Q10 and vitamin E acetate protects against UVB radiation-induced skin injury in mice. Colloids Surf. B Biointerfaces.

[47] Aguida B., Chabi M.M., Baouz S., Mould R., Bell J.D., Pooam M., Andre S., Archambault D., Ahmad M., Jourdan N. (2023). Near-Infrared Light Exposure Triggers ROS to Downregulate Inflammatory Cytokines Induced by SARS-CoV-2 Spike Protein in Human Cell Culture. Antioxidants.

[48] Song H.J., Shin D.U., Eom J.E., Lim K.M., Lim E.Y., Kim Y.I., Kim H.J., Song J.H., Shim M., Choe H. (2023). Artemisia gmelinii Extract Attenuates Particulate Matter-Induced Neutrophilic Inflammation in a Mouse Model of Lung Injury. Antioxidants.

[49] Goodman M., Bostick R.M., Kucuk O., Jones D.P. (2011). Clinical trials of antioxidants as cancer prevention agents: Past, present, and future. Free Radic. Biol. Med..

[50] Higgins M.R., Izadi A., Kaviani M. (2020). Antioxidants and Exercise Performance: With a Focus on Vitamin E and C Supplementation. Int. J. Environ. Res. Public Health.

[51] Sotler R., Poljsak B., Dahmane R., Jukic T., Pavan Jukic D., Rotim C., Trebse P., Starc A. (2019). Prooxidant Activities of Antioxidants and Their Impact on Health. Acta Clin. Croat..

[52] Timoshnikov V.A., Kobzeva T.V., Polyakov N.E., Kontoghiorghes G.J. (2020). Redox Interactions of Vitamin C and Iron: Inhibition of the Pro-Oxidant Activity by Deferiprone. Int. J. Mol. Sci..

[53] Li S., Fasipe B., Laher I. (2022). Potential harms of supplementation with high doses of antioxidants in athletes. J. Exerc. Sci. Fit..

[54] Kordiak J., Bielec F., Jablonski S., Pastuszak-Lewandoska D. (2022). Role of Beta-Carotene in Lung Cancer Primary Chemoprevention: A Systematic Review with Meta-Analysis and Meta-Regression. Nutrients.

[55] Klein E.A., Thompson I.M., Tangen C.M., Crowley J.J., Lucia M.S., Goodman P.J., Minasian L.M., Ford L.G., Parnes H.L., Gaziano J.M. (2011). Vitamin E and the risk of prostate cancer: The Selenium and Vitamin E Cancer Prevention Trial (SELECT). JAMA.

[56] Fuchs-Tarlovsky V. (2013). Role of antioxidants in cancer therapy. Nutrition.

[57] Keppler F., Boros M., Polag D. (2023). Radical-Driven Methane Formation in Humans Evidenced by Exogenous Isotope-Labeled DMSO and Methionine. Antioxidants.

[58] Horvath T., Sandor L., Barath B., Donka T., Barath B., Mohacsi A., Jasz K.D., Hartmann P., Boros M. (2023). Methane Admixture Protects Liver Mitochondria and Improves Graft Function after Static Cold Storage and Reperfusion. Antioxidants.

[59] Zhang W., Zhong R., Qu X., Xiang Y., Ji M. (2023). Effect of 8-Hydroxyguanine DNA Glycosylase 1 on the Function of Immune Cells. Antioxidants.

[60] Arai T., Yoshikai Y., Kamiya J., Nagino M., Uesaka K., Yuasa N., Oda K., Sano T., Nimura Y. (2001). Bilirubin impairs bactericidal activity of neutrophils through an antioxidant mechanism in vitro. J. Surg. Res..

[61] Tsai Y.L., Chen Y., Chen Y.C., Tsai W.C. (2023). KDELC2 Upregulates Glioblastoma Angiogenesis via Reactive Oxygen Species Activation and Tumor-Associated Macrophage Proliferation. Antioxidants.

